# Organoids to model the endometrium: implantation and beyond

**DOI:** 10.1530/RAF-21-0023

**Published:** 2021-08-05

**Authors:** Thomas M Rawlings, Komal Makwana, Maria Tryfonos, Emma S Lucas

**Affiliations:** 1 Division of Biomedical Sciences, Warwick Medical School, University of Warwick, Coventry, UK; 2 Centre for Early Life, Warwick Medical School, University of Warwick, Coventry, UK

**Keywords:** embryo implantation, endometrium, organoid, assembloid

## Abstract

Despite advances in assisted reproductive techniques in the 4 decades since the first human birth after *in vitro* fertilisation, 1–2% of couples experience recurrent implantation failure, and some will never achieve a successful pregnancy even in the absence of a confirmed dysfunction. Furthermore, 1–2% of couples who do conceive, either naturally or with assistance, will experience recurrent early loss of karyotypically normal pregnancies. In both cases, embryo-endometrial interaction is a clear candidate for exploration. The impossibility of studying implantation processes within the human body has necessitated the use of animal models and cell culture approaches. Recent advances in 3-dimensional modelling techniques, namely the advent of organoids, present an exciting opportunity to elucidate the unanswerable within human reproduction. In this review, we will explore the ontogeny of implantation modelling and propose a roadmap to application and discovery.

**Lay summary:**

A significant number of couples experience either recurrent implantation failure or recurrent pregnancy loss. Often, no underlying disorder can be identified. In both cases, the interaction of the embryo and maternal tissues is key. The lining of the womb, the endometrium, becomes receptive to embryo implantation during each menstrual cycle and provides a nourishing and supportive environment to support ongoing pregnancy. It is not possible to study early pregnancy directly, therefore, modelling embryo-endometrium interactions in the laboratory is essential if we wish to understand where this goes wrong. Advances in the lab have resulted in the development of organoids in culture: 3D cellular structures that represent the characteristics of a particular tissue or organ. We describe past and present models of the endometrium and propose a roadmap for future work with organoid models, from fundamental understanding of the endometrial function and implantation processes to the development of therapeutics to improve pregnancy outcomes and gynaecological health.

## Introduction

Although reported success rates in assisted reproduction approach or even surpass natural conceptions, 1–2% of couples will experience recurrent implantation failure, defined as the absence of a positive pregnancy test after three transfers of high-quality embryos (Mascarenhas *et al.* 2021). Some will never achieve a successful pregnancy even in the absence of a confirmed dysfunction. Furthermore, 1–2% of couples who do conceive, either naturally or with assistance, will experience recurrent early loss of karyotypically normal pregnancies with no identifiable cause even after extensive clinical investigation (Bender Atik *et al.* 2018). In both cases, the loss of overtly ‘normal’ embryos suggests that defects of embryo-endometrial interaction might be a plausible explanation.

The purpose of any model system is to generate a physical, theoretical, or mathematical representation of a real phenomenon that is difficult or impractical to observe directly (Rogers 2012), a situation entirely descriptive of the study of human implantation. The ethical and practical impossibility of studying implantation processes within the human body has necessitated the use of animal models and cell culture approaches. These approaches have revealed considerable detail about the requirements for successful implantation but remain far from ideal. Recent advances in 3-dimensional (3D) modelling techniques, namely the advent of organoids, present an exciting opportunity to elucidate the unanswerable within human reproduction. In this review, we will explore the ontogeny of implantation modelling and propose a roadmap to application and discovery.

## The endometrium and implantation

The mucosal lining of the uterus, the endometrium, is a complex tissue comprising luminal and glandular epithelium, as well as stromal, endothelial, and immune cells and provides a nourishing, immune-privileged environment to support successful embryo implantation (Gellersen & Brosens 2014). Formed of two distinct layers, the endometrium undergoes hormone-dependent cyclic renewal with proliferation, differentiation, shedding (menstruation), and regeneration of the upper functional layer in each cycle. Spontaneous, embryo-independent transformation of the tissue, termed decidualisation, occurs in the midluteal phase of each cycle facilitating the development of the decidua of pregnancy to support placentation and foetal development until parturition. Humans are one of only a handful of species in which spontaneous decidualisation has evolved. This is proposed to reflect the need for maternal control over the challenge posed by genetically diverse and mosaic embryos in species where deep haemochorial placentation requires complete maternal investment in the pregnancy (Gellersen & Brosens 2014).

Functional defects in the endometrium, and particularly in the decidualisation process, have been associated with a spectrum of reproductive disorders, ranging from implantation failure and miscarriages to major obstetrical syndromes such as preeclampsia, foetal growth restriction, and preterm birth (reviewed by Lucas *et al.* 2013). However, other than histological descriptions of embryo implantation in hysterectomy samples from the early and mid-20th century (Hertig *et al.* 1956), we know very little about the earliest stages of implantation of the human blastocyst and the pivotal interactions taking place between the midluteal endometrium and the embryo. *In vivo* studies of dynamic implantation processes have been dependent on the use of animal models, while the generation of *in vitro* systems has facilitated the study of human embryo development and endometrial physiology and pathology, albeit largely in isolation. The development of embryo-endometrium co-cultures that faithfully mimic *in vivo* processes will truly unlock the black box of human reproduction.

## Modelling implantation

### Animal models

In the absence of human *in vivo* studies, murine, ovine, and bovine models, among others, have contributed much to the study of endometrial biology and pregnancy. However, the extent to which animal models represent human physiology is limited due to marked interspecies differences in reproductive strategy, implantation, and placentation. These have been reviewed extensively elsewhere (Lee & DeMayo 2004, Carter 2007, 2020, Morrison *et al.* 2018).

Livestock models have been utilised to pioneer many techniques used in modern assisted reproductive technologies (ART) (reviewed by Morrison *et al.* 2018). Sheep and cattle have been used to model human pregnancy while also advancing their commercial productivity with similar goals to human ART: to improve conception and live birth rates and reduce the incidence of pregnancy loss. Considerable differences in maternal recognition of pregnancy, implantation timing, and placental morphology make direct inference to human implantation difficult, nonetheless, conserved mechanisms are present (Tinning *et al.* 2020). Notwithstanding the value of sheep to model human foetal development (Morrison *et al.* 2018), the utility of large domestic animals to model early human pregnancy is limited (Carter 2020). Mouse models have been used to explore all aspects of reproduction, including implantation and the establishment of pregnancy. The mouse has been a preferred model for researchers for a number of reasons including litter-bearing status, rapid conception and gestation, haemochorial placentation, and genetic tractability; all of which provide an abundance of material for study and the ability to target specific pathways of interest to determine the impact on fecundity and fertility (Carter 2020). However, progesterone receptor expression dynamics vary greatly between humans and mice (Teilmann *et al.* 2006), and decidualisation in mouse is dependent on an embryonic stimulus (Lopes *et al.* 2004) rather than a spontaneous cyclic process. Overall, despite the many advances in our understanding of early pregnancy from animal models, interspecies differences have hindered the translation of understanding to human embryo implantation research.

### *In vitro* models

**Figure 1:**
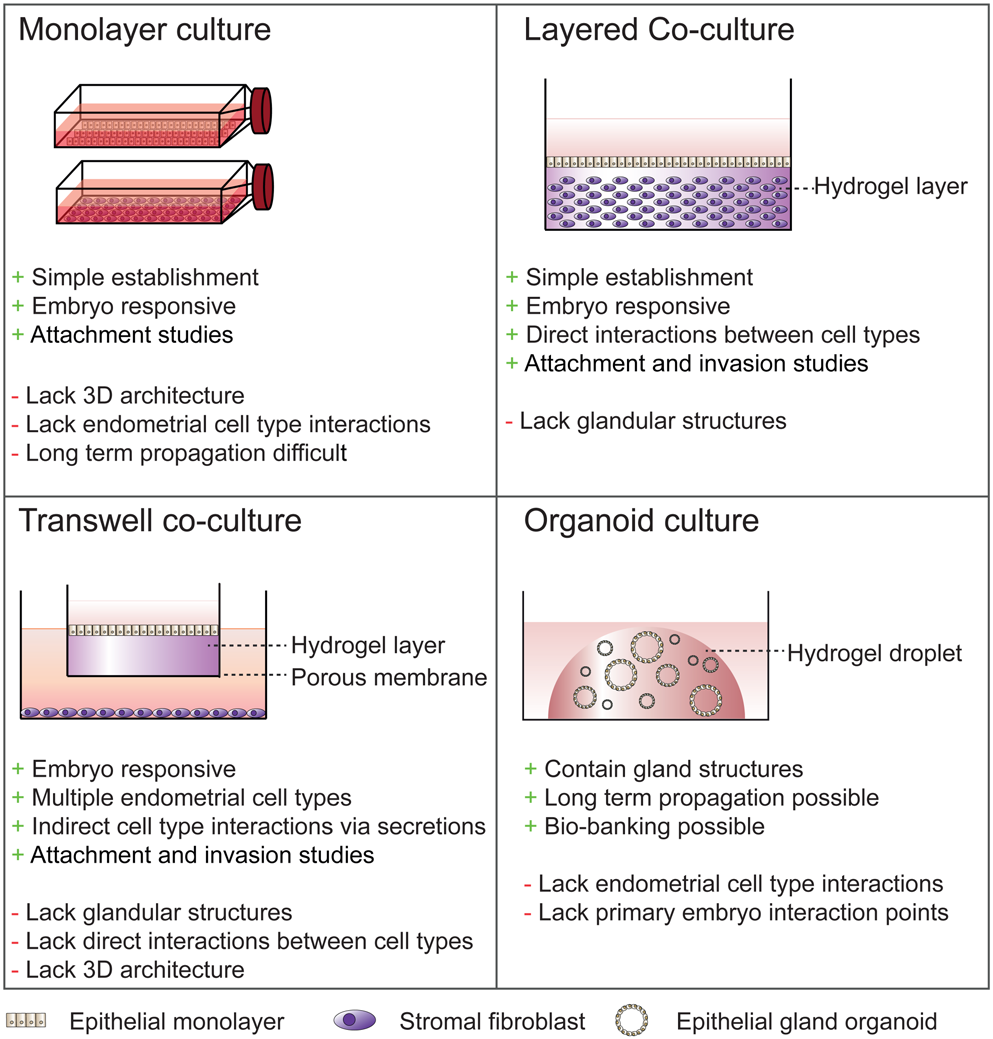
*In vitro* endometrial models to study implantation. Schematic representations of existing models for modelling endometrium-embryo interactions. The main benefits (**+**) and shortfalls (**-**) of each system are indicated.

To circumvent interspecies variability at implantation, *in vitro* models have been developed using primary endometrial epithelial or stromal cell cultures as well as endometrium-derived cell lines. *In vitro* endometrial models aim to represent at least certain aspects of the human implantation environment while retaining levels of experimental control and manipulability (Fig. 1 and Table 1). In combination with a range of molecular techniques and other assays, these models present effective tools to close the knowledge gap regarding human embryo implantation and answer a diverse panel of questions.

**Table 1:** *In vitro* models to study human endometrium-embryo interaction.

Model format/cell types	Embryo/spheroid	Measures of interaction				References
		Embryo/spheroid	Stromal cells	Epithelial cells	Trophoblast	
Monolayer						
Primary human endometrial stromal cells	Hatched human blastocysts, high- and low-quality human embryos, human embryo-conditioned media, mouse blastocysts, human trophoblast spheroids, embryonic stem cell-derived trophoblast spheroids	Attachment (apposition and anchoring), trophoblast outgrowth and invasion, hCG secretion	Cell migration, secretion of implantation-associated cytokines, gene expression profiles, calcium signalling, ‘biosensing’ embryo quality			Carver *et al.* (2003), Grewal *et al.* (2008, 2010), Teklenburg *et al.* (2010), Weimar *et al.* (2012), Brosens *et al.* (2014), Lee *et al.* (2015), Berkhout *et al.* (2018)
Human endometrial stromal cell lines (T-HESC; hTERT-immortalised endometrial stromal cells)	Human trophoblast spheroids (AC-1M88, Sw.71)	*Attachment, expansion, outgrowth and invasion, CEACAM1 expression	Mmigration			Holmberg * et al.* (2012), Gellersen *et al.* (2013)
Primary human endometrial epithelial cells	Cleavage stage human embryos and blastocysts, human embryo-conditioned media, mouse blastocysts, human trophoblast spheroids (trophoblast stem cell-, embryonic stem cell-and/or choriocarcinoma -derived)	Blastocyst rate and quality, blastocyst adhesion, spheroid attachment and outgrowth		Gene expression and secretion of implantation-associated chemokines, chemokine receptor expression and localisation, gene expression profiles, morphological assessment		Lindenberg *et al.* (1985), Simón *et al.* (1997, 1999), Meseguer *et al.* (2001), Caballero-Campo *et al.* (2002), Dominguez *et al.* (2003), Lee *et al.* (2015), Le Saint *et al.* (2019), Evans *et al.* (2020).
Human endometrial epithelial cell lines (Ishikawa, RL95-2)	Hatched and unhatched human blastocysts, hatched mouse blastocysts, human trophoblast spheroids (trophoblast stem cell-, embryonic stem cell-and/or choriocarcinoma -derived)	Attachment and outgrowth/invasion		Gene expression profiles		Lee *et al.* (2015), Huang *et al.* (2017), Berneau *et al.* (2019), Evans *et al.* (2020), Ruane *et al.* (2020).
Layered culture						
Primary human endometrial epithelial and stromal cells	Expanded/hatching human blastocysts, trophoblast spheroids (choriocarcinoma, JAr)	Embryo attachment, trophoblast invasion and syncytium formation, spheroid attachment and outgrowth/invasion				Bentin-Ley *et al.* (2000), Wang * et al.* (2012)
Human endometrial epithelial cell lines (RL95-2, HEC-1A, Ishikawa), immortalised and primary human endometrial stromal cells	Trophoblast spheroids (Jar choriocarcinoma)	*Attachment and outgrowth/invasion				Evron *et al.* (2011), Wang * et al.* (2012)
Transwell culture						
Primary human endometrial stromal cells and immortalised cell lines (St-T1b; T-HESC)	Trophoblast cells (AC-1M88)		Mmotility and invasion		Invasiveness	Gellersen *et al.* (2010, 2013)
Organoids						
Primary human endometrial epithelial cells	Embryo conditioned media			Pinopode development, glycodelin secretion profile.		Luddi *et al.* (2021)
Assembloid						
Primary human endometrial epithelial and stromal cells	Hatched human blastocysts	^†^Expansion and attachment; EM cells: morphology and motility				Rawlings *et al.* (2021)

*Spheroid; ^†^Embryos.

EM cells, endometrial cells.

#### Monolayer cultures

Two-dimensional (2D) cultures of endometrial stromal and epithelial cells have both been utilised to investigate implantation processes (Fig. 1). Since the first report using monolayer epithelial cells to describe interactions between the luminal epithelium and an implanting embryo (Lindenberg *et al.* 1985), many studies have used this approach to study early feto-maternal interactions (Weimar *et al.* 2013, Aplin & Ruane 2017). In response to embryonic signals, co-cultured epithelial cells upregulate expression of the receptivity genes CXC chemokine receptor type 1 (CXCR1), CXCR4, and CXCR5, and the chemokine interleukin (IL)-8 (Caballero-Campo *et al.* 2002, Dominguez *et al.* 2003), encoded by *CXCL8*, as well as epithelial cell surface molecules implicated in embryo implantation, such as mucin 1 (MUC1) (Simón *et al.* 1997, Meseguer *et al.* 2001) and osteopontin (OPN) (von Wolff *et al.* 2001, Erikson *et al.* 2009, Berneau *et al.* 2019) (Table 1). Co-culture of *in vitro* fertilised (IVF) embryos with an autologous epithelial monolayer prior to embryo transfer increased the frequency of high-quality blastocyst formation and implantation rates (Simón *et al.* 1999, Le Saint *et al.* 2019). However, a significant benefit of the co-culture to live birth rates remains to be confirmed (Le Saint *et al.* 2019). Trophoblast spheroids, as a surrogate for human blastocysts, have also been used successfully in co-culture with epithelial monolayers to investigate embryo attachment to the luminal epithelium (Weimar *et al.* 2013, Lee *et al.* 2015, Huang *et al.* 2017, Evans *et al.* 2020, Ruane *et al.* 2020) (Table 1). These studies demonstrate the reciprocal relationship between luminal epithelium and preimplantation embryos in preparation for human implantation. However, endometrial epithelial cells are technically challenging to propagate as monolayers and cannot be cultured long-term (Varma *et al.* 1982, Fitzgerald *et al.* 2021). They also do not represent the normal physiology and architecture of glands *in vivo* (Gray *et al.* 2001).

Primary human endometrial stromal cells have been used extensively to study human decidualisation and the peri-implantation endometrial environment. Studies of primary stromal monolayers have demonstrated embryo-stroma interactions and the retention of patient phenotypes by these cells. Carver and colleagues (2003) co-cultured stromal cell monolayers with hatched day five human blastocysts for 3 days and demonstrated that blastocysts could attach to and invade a decidualised stromal monolayer but did not interact with undifferentiated cells. In midluteal endometrium, decidualising stromal cells transiently attain a myo-fibroblastic phenotype, in which they become migratory and can produce extracellular matrix (ECM)-degrading matrix metallopeptidases (MMP) (Anacker *et al.* 2011), a process which is essential to allow trophoblast outgrowth (Grewal *et al.* 2008, 2010). Decidualised stromal cells are sensitive to embryo quality, selectively migrating towards high-quality human embryos but downregulating implantation-associated genes and the migratory phenotype in the presence of poor-quality or arresting embryos or conditioned media (Teklenburg *et al.* 2010, Weimar *et al.* 2012, Brosens *et al.* 2014, Berkhout *et al.* 2018) (Table 1).

Monolayer stromal co-cultures with blastocysts have also been used to investigate endometrial function and dysfunction in reproductive failure. Decidualising stromal cells of recurrent pregnancy loss (RPL) patients fail to inhibit implantation genes when co-cultured with poor-quality embryos (Weimar *et al.* 2012). This suggests a defect in embryo quality ‘biosensing’ in the endometrium of these women, manifesting as a loss of embryo selectivity. Also referred to as the ‘selection failure hypothesis, this failure to prevent implantation of poor-quality embryos is predicted to lead to subsequent miscarriage (Aplin *et al.* 1996, Macklon & Brosens 2014).

Overall, monolayer endometrial cell models provide a simple and robust model for studying early embryo implantation and embryo-endometrial interactions in humans. A major disadvantage of monolayer models is the restriction to a single endometrial cell type, disregarding stromal-epithelial interactions within the *in vivo* environment, as well as the roles of resident immune and endothelial cell populations. Additionally, although these models have been utilised to study the initial attachment and invasion of embryos, they lack the 3D architecture essential to study trophoblast invasion and the foundations of placentation.

#### Layered co-cultures

Glandular development and differentiation are dependent on stromal secretions (reviewed by Fitzgerald *et al.* 2021), exemplifying the dependence of the peri-implantation endometrial environment on synchronous paracrine signals, cell–cell, and cell-matrix interactions between multiple cell types of the decidualising endometrium. To study these interactions, more complex co-culture models have been developed (Fig. 1 and Table 1) and these will be explored briefly here.

To establish 3D cultures, cells are seeded into or on top of a scaffold to mimic their spatial environment within the structure of the ECM. The most common scaffold is the hydrogel, a hydrophilic polymer chain network that can be easily fine-tuned to mimic the structural properties of a native tissue ECM. Layered co-cultures of endometrial epithelial cells grown over a stromal cell-containing collagen hydrogel resemble a simplified human endometrium and aim to replicate the complexity of the *in vivo* endometrium more closely (Bentin-Ley *et al.* 1994, Evron *et al.* 2011, Wang *et al.* 2012). Luminal epithelium polarisation and embryo attachment within 48 h were revealed in one such model by scanning electron microscopy, with apparent penetration of syncytiotrophoblast through the epithelium into the underlying stromal layer (Bentin-Ley *et al.* 2000). In place of embryos, trophoblast cell line spheroids, such as the JAr cell line (John *et al.* 1993), have also been utilised to study implantation in layered co-culture models (Evron *et al.* 2011, Wang *et al.* 2012). The attachment of trophoblast spheroids to epithelial cell lines was improved by co-culturing with stromal cells (Wang *et al.* 2012) and dependent on the receptivity status of the stromal cells (Evron *et al.* 2011). Occasional spontaneous gland-like structures were observed in these cultures (Wang *et al.* 2012) although layered models generally fail to form glandular structures reproducibly and thus while useful for studying very early implantation processes, are not truly representative of the tissue at implantation.

Transwell co-culture models enhanced the ability to study trophoblast invasion: using dual-chambered systems containing an extracellular matrix (Matrigel™)-coated porous filter insert, adherent trophoblast cells can invade the matrix and migrate through the filter (Aplin 2006, Lash *et al.* 2007) (Fig. 1). By seeding endometrial epithelial cells on top of the Matrigel™ in the transwell insert and culturing stromal cells in the well beneath the filter (Arnold *et al.* 2001, Pierro *et al.* 2001, Bläuer *et al.* 2005), the transwell approach provides an easily manipulatable assay to assess trophoblast invasion in the presence of endometrial cells. The presence of trophoblasts also improves the invasiveness of decidualised stromal cells in transwell systems (Gellersen *et al.* 2010, 2013). However, cell–cell contact between epithelial and stromal cells is not established in these models and so critical interactions to orchestrate trophoblast invasion and endometrial tissue remodelling are likely to be missing.

Clearly, layered and transwell co-culture approaches improve upon monolayer cultures by incorporating both stromal and epithelial cells into a 3D space, providing the architecture for a more physiological model to study implantation. However, these models conspicuously lack glandular structures, a functional unit of the endometrium essential for embryo implantation.

## Modelling endometrial glands: the missing link?

Secretory transformation of endometrial glands results in the production of histotroph, or ‘uterine milk’. Histotroph composition has been studied extensively during the menstrual cycle, consisting largely of glycogen and glycoproteins, as well as other components such as amino acids and lipid droplets (Burton *et al.* 2002). This rich secreted product provides nutrition for the embryo until the onset of placental perfusion (Burton *et al.* 2007, 2010). Studies using animal models, such as the uterine gland knockout sheep, produced by a progestin-induced gland knock out, demonstrate that without endometrial glands, embryo implantation is inhibited (Gray *et al.* 2001). Progestin uterine gland knockout mice also exhibit implantation failure, with reduced expression of key implantation genes (Kelleher *et al.* 2016).

**Figure 2:**
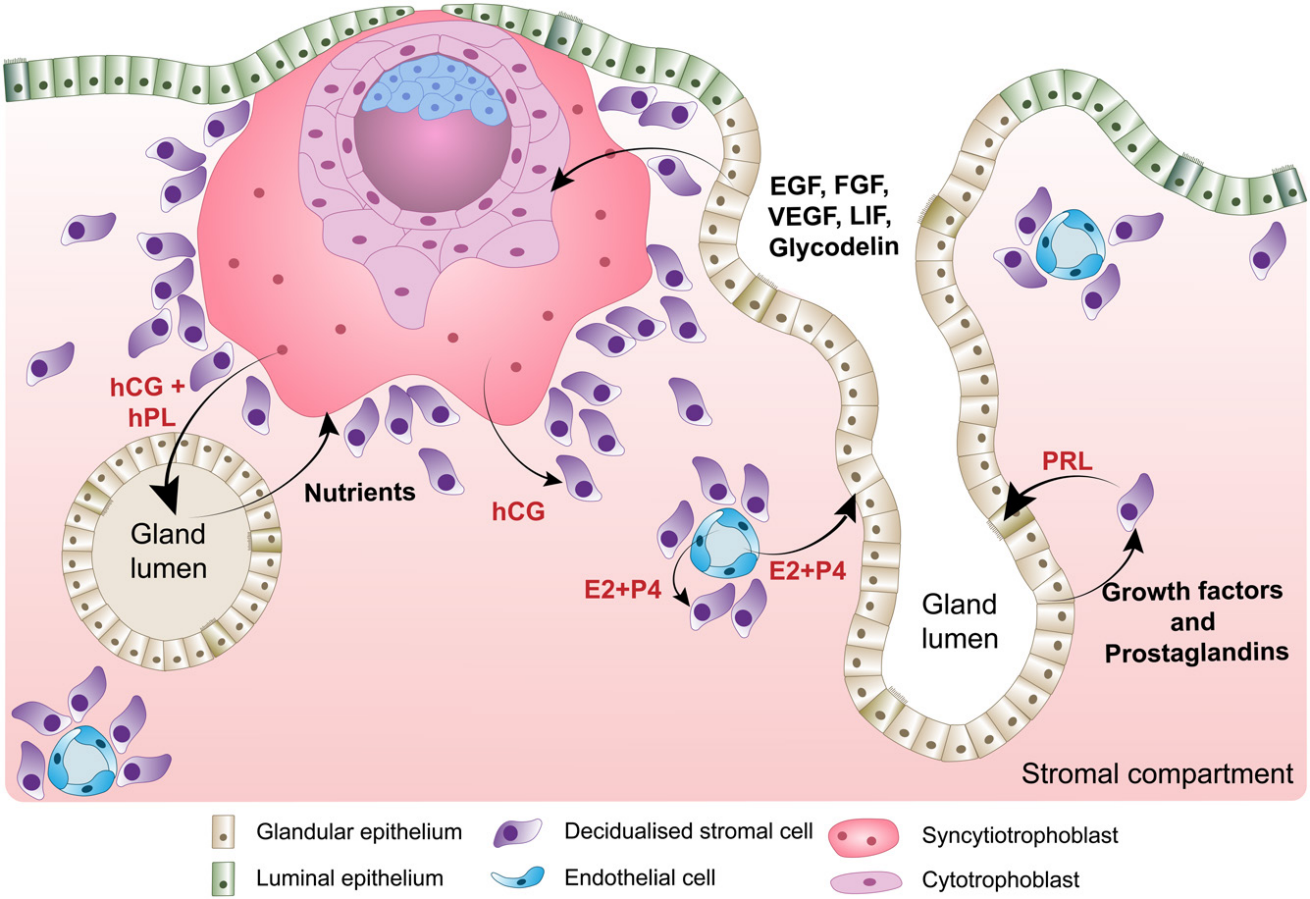
Embryo-endometrium crosstalk at implantation. Schematic representation of the crosstalk between endometrial glands, decidualised stromal cells, corpus luteum, and trophoblasts cells of the invading embryo. During pregnancy, the syncytiotrophoblast cells produce hCG to maintain the corpus luteum so progesterone can be continually produced. Progesterone is essential for the maintenance of the decidualised cell and differentiated glands. A key secretion of the decidual cells at implantation is PRL, which acts on the glands through PRLR to maintain the glandular secretory phenotype. Additionally, trophoblast cells act directly on the glands through hCG and hPL secretion, resulting in the production of growth factors, nutrients, and receptivity markers such as LIF and glycodelin. In turn, these factors improve trophoblast invasion, thus forming a positive feedback loop.

Besides providing nutritional support to the implanting embryo, endometrial glands upregulate genes required for embryo receptivity and implantation, including numerous secreted factors essential for implantation (Fig. 2). For example, osteopontin (OPN), encoded by phosphoprotein 1 (*SPP1*), contains the integrin-binding motif RGD, a tripeptide of amino acids arginine, glycine, and aspartate, which facilitates embryo adhesion to the luminal epithelium (Singh & Aplin 2009, Berneau *et al.* 2019). Leukaemia inhibitory factor, encoded by *LIF*, is a protein related to blastocyst adhesion, as well as having roles in embryonic development and trophoblast differentiation (Salleh & Giribabu 2014). Glycodelin, a dimeric glycoprotein, encoded by progestogen-associated endometrial protein (*PAEP*), also participates in interactions between the implanting blastocyst and luminal epithelium. *In vitro*, induction of glycodelin secretion improves trophoblast spheroid attachment while silencing *PAEP* inhibits attachment (Uchida *et al.* 2007, So *et al.* 2012).

Human endometrial glands also secrete the growth factors EGF, vascular endothelial growth factor (VEGF), and transforming growth factor-beta (TGFB), the cognate receptors for which are expressed by the trophectoderm of the implanting blastocyst. EGF stimulates proliferation of cytotrophoblast and secretion of human chorionic gonadotropin (hCG) and human placental lactogen (hPL) by the syncytiotrophoblast (Ladines-Llave *et al.* 1991, Maruo *et al.* 1997) (Fig. 2). VEGF enhances the adhesion of the trophoblast to the luminal epithelium (Binder *et al.* 2014), while TGFB increases ECM remodelling, via the secretion of fibronectin, for example, thereby facilitating endometrial adhesion for invading trophoblasts (Feinberg *et al.* 1994).

Overall, the glands and their secretions are essential for implantation, and therefore, being able to model the glands *in vitro* is an essential step towards studying and understanding human embryo implantation and early pregnancy events. Recent development of glandular organoid models may have brought us closer than ever to establish a physiological model of the human endometrium.

## Organoids of the reproductive system

‘Organoid’ describes 3D structures that resemble organs or tissues *ex vivo* in a supportive hydrogel droplet, such as Matrigel™, replacing the ECM of the originating tissue. Organoids arise from single cells or small clusters of cells with stem or progenitor properties in cultures with complex growth media designed to mimic organ- or tissue-specific signalling pathways and recapitulate the niche environment (Kim *et al.* 2020*b*). Organoids differ from previous *in vitro* model systems in that they spontaneously organise into architectures that resemble their corresponding *in vivo* tissue or organ in a culture plate. Additionally, organoid models functionally mimic their tissue of origin and can recapitulate developmental processes that take place *in vivo*, thus allowing the study of developing and differentiating cells in real time. Growth factor and inhibitor combinations in these complex media support proliferation and renewal as well as differentiation and substitute for the absence of the tissue complexity present *in vivo*. Most reported models employ a consistent core set of factors that are reviewed elsewhere (Kretzschmar & Clevers 2016, Alzamil *et al.* 2021). Organoids have been generated from the vast majority of endoderm-derived tissues including the colon (Sato *et al.* 2011), intestine (Fujii *et al.* 2018), stomach (Bartfeld *et al.* 2015, Schlaermann *et al.* 2016), liver (Huch *et al.* 2015, Hu *et al.* 2018), pancreas (Loomans *et al.* 2018), lung/airway (Sachs *et al.* 2019) and bladder (Lee *et al.* 2018).

**Figure 3:**
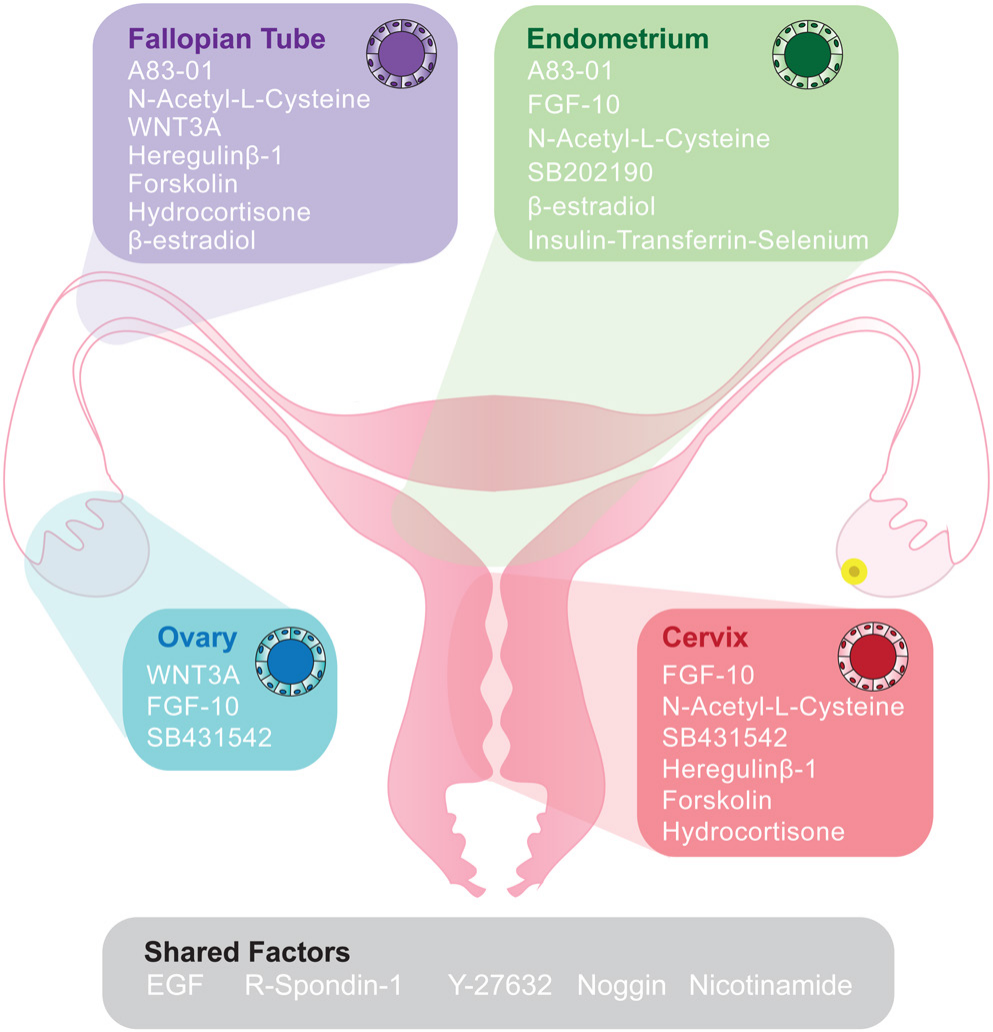
Organoid models of the female reproductive tract. In the human female reproductive tract, organoid models have been derived from the endometrium, fallopian tube, ovary, and cervix tissues and recapitulate the epithelial structure of the tissue of origin. All systems have been cultured in basal medium including Advanced DMEM/F12, B-27, and N-2 supplements, antibiotics, and L-Glutamine but also include a cocktail of growth factors and inhibitors to promote proliferation and maintain undifferentiated or progenitor cell-like conditions. Common to all models is the growth factor EGF and the WNT signalling activator R-Spondin-1, as well as the ROCK inhibitor Y-27632 and BMP pathway inhibitor Noggin, and Nicotinamide, a survival factor, and ROCK inhibitor. Other factors which are often added include the TGFB receptor inhibitor A83-01, the activin/ BMP/TGFB inhibitor, SB431542, and the p38 MAPK inhibitor SB202190; Growth factors FGF-10 (fibroblast growth factor) and WNT3A as well as other factors such as N-acetyl-L-cysteine (antioxidant), β-estradiol (mitogen), heregulin-β (growth factor), forskolin (adenylyl cyclase activator), and hydrocortisone (glucocorticoid).

Several recent reports have demonstrated progress in modelling human reproductive epithelia using organoid systems. In the female reproductive system, endometrium (Boretto *et al.* 2017, Turco *et al.* 2017), fallopian tube (Kessler *et al.* 2015), cervix (Chumduri *et al.* 20^21^, Maru *et al.* 2020, Lohmussaar *et al.* 2021), and ovarian cancer (Kopper *et al.* 2019, Maenhoudt *et al.* 2020) models have been described (Fig. 3), as well as mammary gland (Linnemann *et al.* 2015). These models facilitate the study of the general biology and histology of the reproductive tissues since they recapitulate epithelial cell morphology, function, and cellular heterogeneity while being genetically stable. These organoids are hormone-responsive and can be differentiated under specific conditions (reviewed by Alzamil *et al.* 2021). In the male reproductive tract, organoid systems are described for both testis (Alves-Lopes *et al.* 2017, Baert *et al.* 2017, Pendergraft *et al.* 2017) and prostate (Chua *et al.* 2014, Karthaus *et al.* 2014). Trophoblast organoids to model placental development (Haider *et al.* 2018, Turco *et al.* 2018), as well as embryo-like organoids, known as blastoids have also been described (Zheng *et al.* 2019, Liu *et al.* 2021, Yu *et al.* 2021). Human blastoids recapitulate the key morphology of preimplantation blastocysts, including cell-lineage composition and allocation, with transcriptomic similarities but lack important structures like the zona pellucida (Liu *et al.* 2021, Yu *et al.* 2021). Blastoids and trophoblast organoids both offer the opportunity to study implantation and early developmental processes at a greater scale than possible with research embryos, and in cases where embryos are unavailable for research use or such applications are prohibited.

The emergence of these organoid models for human reproduction and fertility represents a huge leap forward for the field, presenting a considerable opportunity to advance our knowledge of many ‘hidden’ processes within these systems. Limitations remain, for example, ease of accessibility means comparisons with healthy tissue are limited in some cases, while optimisation of other models is still required.

### Endometrial gland organoids

Organoid-like structures established from endometrial epithelial cells were first reported in 1988, derived from primary gland fragments seeded into Matrigel™ (Rinehart *et al.* 1988). Glandular structures were retained after tissue digest collapsed and monolayer colony outgrowth was observed within 7 to 10 days. Following several weeks of culture, these colonies formed large cystic organoid structures of polarised columnar epithelial cells with luminal microvilli and could be maintained in culture for at least 6 months. Endometrial gland organoids were also produced in Matrigel™ and grown in a transwell system, in co-culture with stromal cells grown in the lower chamber (Bläuer *et al.* 2005). In this system, the organoids were responsive to oestradiol treatment in the presence of the stromal cell layer. These reports failed to recapitulate the gland phenotype fully, and genetic stability was not confirmed, nor were the culture conditions chemically defined. Renewed interest in endometrial gland organoids was stimulated recently by parallel reports delivering a step-change in our ability to study the function of these structures (Boretto *et al.* 2017, Turco *et al.* 2017) (Fig. 3). The resulting organoids are genetically stable, have the clonogenic capacity, cellular heterogeneity, and can recapitulate molecular and histological similarities of the glands *in vivo* (Boretto *et al.* 2017, Turco *et al.* 2017, Fitzgerald *et al.* 2019). They can be derived from physiologically diverse endometrial tissues throughout the menstrual cycle, after menopause, and from decidual pregnancy samples (Turco *et al.* 2017). Like other organoid models, endometrial gland organoids are cultured in Matrigel™ and are dependent on a chemically defined complex ‘expansion medium’ containing a variety of growth factors required to promote proliferation and inhibit differentiation, including fibroblast growth factor 10 (FGF10), EGF, Wingless and Int1 (WNT) signalling activator R-spondin 1 (RSPO1), bone morphogenetic protein (BMP) inhibitor Noggin, and the TGFβ antagonist, A83-01. Unlike the initial report (Rinehart *et al.* 1988), these recent models see rapid organoid formation from gland fragments within 7–10 days. Oestrogen treatment induces proliferation of the cells with an increase in Ki67+ cells (Boretto *et al.* 2017) as well as stimulating ciliogenesis (Haider *et al.* 2019) and, when combined with progesterone, secretion of glycodelin-A corresponds to tissue profiles in mid-secretory endometrium (Luddi *et al.* 2020). Glycodelin profiles were further modulated after incubation of organoids with embryo-conditioned medium (Luddi *et al.* 2021), confirming embryo-endometrial crosstalk is possible within the model (Table 1). Metabolomic analysis of the secretome of endometrial organoids also demonstrates unique characteristics of the apical (inter-organoid) and basolateral (extra-organoid) secretory profiles, characteristic of that predicted *in vivo* (Simintiras *et al.* 2021). Through exposure to pregnancy signals of the stroma (cyclic AMP and prolactin (PRL)) and trophoblast (hCG and hPL), the organoids acquired a decidual-like phenotype akin to early pregnancy (Turco *et al.* 2017).

Thus far, endometrial gland organoid models are still in their infancy, albeit one that surpasses prior systems physiologically. The opportunity to address questions of normal endometrial physiology and implantation processes, therefore, lies along our path.

## Endometrial gland organoids and the future: a roadmap to utility

### Endometrial gland organoids, assembloids, and modelling embryo implantation

Despite their structural and functional recapitulation of endometrial gland phenotypes, endometrial organoid models do not faithfully represent the midluteal endometrium, due to being grown in isolation without the influence of other endometrial cell types present in the tissue. Endometrial stromal cells are essential for glandular regeneration, expansion, and differentiation throughout the menstrual cycle. For example, through the secretion of PRL, decidualising stroma regulates gland differentiation via the PRL receptor (PRLR), whose expression peaks in the endometrial glands during the mid-secretory phase (Jones *et al.* 1998). PRL induces glandular differentiation by stimulating the Janus kinase (JAK)/STATs and mitogen-activated protein kinase (MAPK) pathways. In parallel, apoptosis in the glandular epithelium is inhibited by activation of the phosphatidylinositol 3 kinase (PI3K) pathway (Jabbour *et al.* 2002).

Recently, several protocols have described stromal-epithelial co-cultures retaining physiological structure. Slices of full thickness endometrial tissue cultured in collagen gel maintain true histoarchitecture of the endometrium, and a decidual response similar to the *in vivo* profile can be induced following differentiation with oestradiol (E2) and progesterone (P4) (Muruganandan *et al.* 2020). Others have reported the use of porous 3D scaffolds to establish co-cultures. Using a collagen-based scaffold, Abbas and colleagues demonstrated that stromal cells were able to proliferate within the scaffold pores and deposit their own ECM, while fragments of endometrial gland organoids seeded on top of the scaffolds formed a luminal epithelium reminiscent of the tissue structure *in vivo* (Abbas *et al.* 2020). As well as solid scaffolds, synthetic hydrogels, such as polyethylene glycol (PEG), functionalised with ECM- and integrin-binding peptides have been utilised as a replacement for animal-derived hydrogels. When cultured in PEG hydrogels, endometrial stromal cells proliferate and decidualise in response to hormonal stimulation (Cook *et al.* 2017). Modulating the PEG hydrogel altered cell behaviour, highlighting the importance of matrix conditions when developing a representative model of the endometrium (Cook *et al.* 2017). Finally, another recent protocol reported self-aggregation of epithelial and stromal cells in a scaffold-free environment to form structures containing a stromal cell centre and an outer layer of epithelial cells within an agarose mould (Wiwatpanit *et al.* 2020).

An alternative approach to produce a structurally and functionally physiological model of the endometrium is to incorporate endometrial stromal cells into endometrial organoid cultures. Co-culturing of organoids with stromal cells has been reported in other organoid systems, such as the bladder and brain, and these models are designated as ‘assembloids’ (Kim *et al.* 2020*a*, Andersen *et al.* 2020, Miura *et al.* 2020). In the case of bladder, the applicability of organoids to *in vivo* pathology and disease is uncertain because, in isolation, they do not recapitulate the native tissue architecture and microenvironment. To overcome these limitations, bladder organoids were mixed with components of the bladder stroma, such as stromal, endothelial, and immune cells, and a muscle layer to form bladder assembloids (Kim *et al.* 2020). The assembloids represent a more physiological model of the native organ or tissue, and therefore, should provide a more faithful model for testing drug responses and mimicking disease phenotypes.

**Figure 4:**
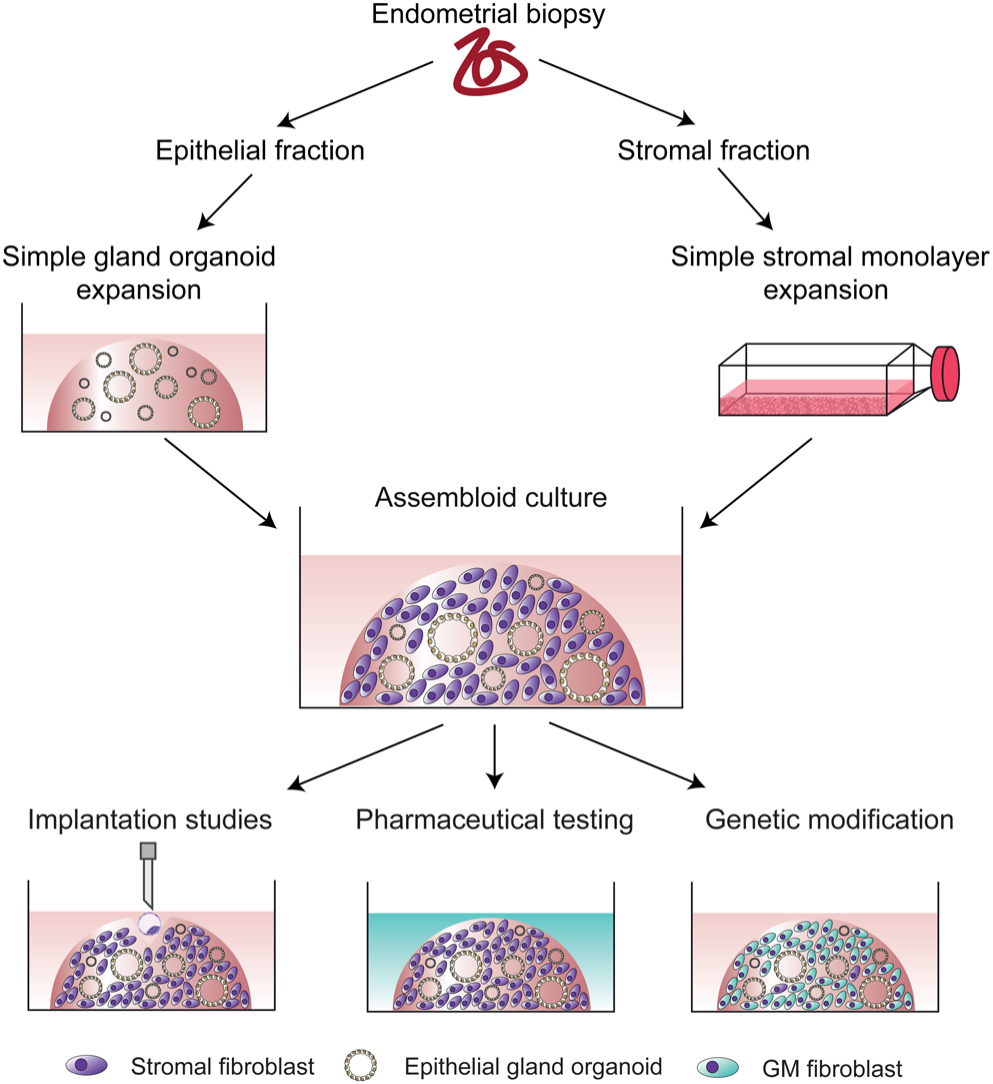
Establishment of an endometrial assembloid model. Schematic representation of the establishment of the endometrial assembloid model. Endometrial pipelle biopsies are digested and separated into epithelial and stromal fractions. Epithelial cells are expanded in Matrigel using the simple gland organoid culture approach for two passages, while stromal cells are expanded in monolayer culture. At passage 2, digested organoids and stromal cells are combined and encapsulated in collagen hydrogel to form an assembloid culture which then follows growth and differentiation protocols as required. Current and future applications for the assembloids include, but are not limited to embryo implantation studies, pharmaceutical testing (e.g. drugs or small molecules) and genetic modification of the different cell populations.

Recently, we have reported the establishment of endometrial assembloids whereby the gland organoid model was modified to incorporate stromal cells (Rawlings *et al.* 2021). Gland organoids were expanded from primary epithelial cells while in parallel the stromal fraction was propagated by standard monolayer culture (Fig. 4). Single-cell stromal suspensions were combined with manually digested organoids, seeded into a collagen hydrogel, and cultured in an expansion medium supplemented with E2. In this model, gland organoid formation was unperturbed by stromal co-culture, and assembloids resemble the architecture of native endometrium more closely than the gland organoids alone. The addition of stromal cells should abrogate the need for many components of the complex medium of growth factors and inhibitors to maintain secretory gland organoids, and indeed a minimal differentiation medium supported robust glandular differentiation in the assembloid model (Rawlings *et al.* 2021). Based on single-cell transcriptomic analysis, decidualised assembloids closely resemble the mid-secretory endometrium, containing several subpopulations of both stromal and epithelial cells, including senescent stromal and epithelial cells, which secrete many canonical implantation factors. Co-culturing human blastocysts with decidualised assembloids demonstrated that in the presence of senescent decidual cells, the assembloids engender a dynamic implantation environment, enabling embryo expansion and attachment, although persistent endometrial senescence led to the gradual disintegration of the assembloid matrix, likely through the actions of MMPs (Freitas-Rodriguez *et al.* 2017). Pharmacological inhibition of stress responses in pre-decidual cells by a tyrosine kinase inhibitor inhibited the propagation of decidual senescence, impeding assembloid breakdown. However, the lack of senescent cells resulted in the entrapment of the blastocysts in the largely static assembloid matrix. This study not only demonstrated that the endometrial assembloid model could be used as a novel embryo implantation model but also confirms previous reports that decidual senescence controls endometrial fate decisions during implantation and early pregnancy (Brighton *et al.* 2017, Lucas *et al.* 2020). Challenges for the endometrial assembloid model are to introduce a luminal epithelium, in order to study sequential blastocyst attachment and invasion, as well as the incorporation of immune cells such as uterine natural killer cells to mimic tissue remodelling processes predicted to take place during implantation (Brighton *et al.* 2017, Kong *et al.* 2021). Moreover, long-term maintenance and propagation of endometrial assembloids to mimic the cycling tissue have yet to be developed.

The major challenge in increasing complexity for a physiological model of the endometrium suitable for use in implantation studies is being able to balance the needs of different cell types within the model. For example, the basement membrane preparation Matrigel™, used frequently for organoid culture, is unsuitable as an extracellular matrix for stromal cells (Arnold *et al.* 2001). Beyond the co-culture of stromal cells with epithelial organoids, endothelial cells may be required to directly invade trophoblasts for placentation (Weiss *et al.* 2016). Additionally, immune cells, such as resident uterine natural killer cells, have been demonstrated to be essential for providing a robust decidual matrix suitable for successful embryo implantation (Kong *et al.* 2021). A microfluidic approach may be the solution to this potential dilemma.

### Endometrial pathologies, genetic manipulation, and biomarker discovery

**Figure 5:**
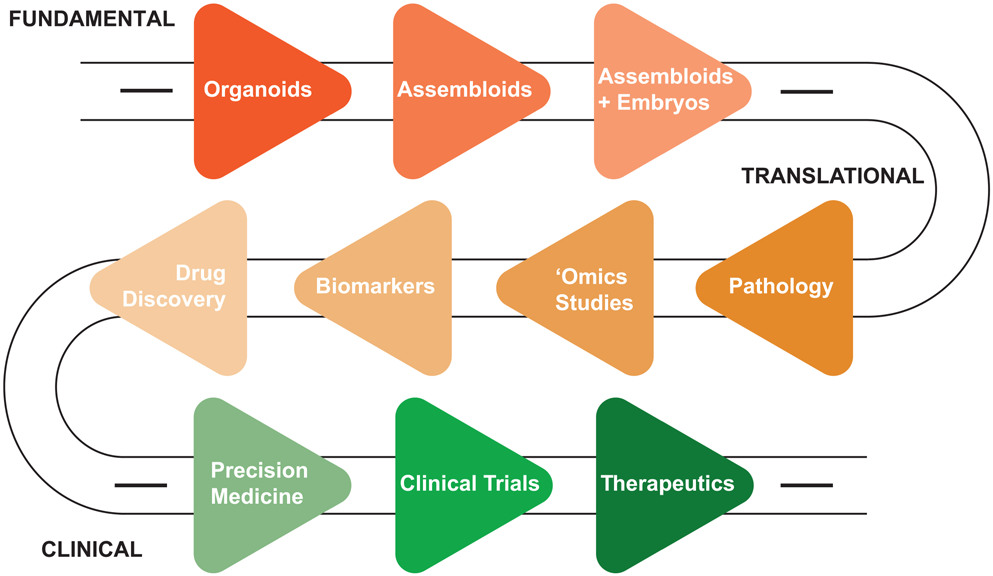
A roadmap to application for endometrial organoids. Schematic representation of the proposed roadmap to application for the endometrial organoids. The roadmap is divided into fundamental (red), translational (orange) and clinical (green) approaches to implantation research. This roadmap presents the trajectory from defining the embryo-endometrial interactions during and after implantation to disease modelling and biomarker- and drug- discovery, through developing successful therapeutic interventions for pathologies that impede successful pregnancy.

Beyond understanding the fundamentals of embryo implantation, endometrial pathologies that impede fertility must also be studied to advance the provision of therapeutics (Fig. 5). The development of medical treatments for many human diseases is limited by patient variation, difficulties in predicting outcomes, and lengthy preclinical drug testing. This is restricted further for applications in human reproduction by our limited ability to study *in vivo* processes in normal tissues. Therefore, organoid and assembloid cultures based on specific pathologies and even on individual patients are expected to develop into powerful tools for precision therapy, permitting a personalised approach to drug discovery, development, and screening. Primary cancers, infectious diseases, and developmental diseases can be replicated with *ex vivo* biopsy samples from patients. Testing prospective pharmacological treatments on human organoids could facilitate the identification of patient group-specific responses and lead to targeted therapeutic approaches. Indeed, initial explorations into the utility of gland organoids in studying endometrial pathology have been reported, demonstrating the value of patient-specific cultures to study pathologies such as endometriosis and cancer (Boretto *et al.* 2017, 2019).

Endometriosis is an endometrial disorder characterised by the ectopic growth of endometrial tissue. Endometriosis is associated with infertility and affects 10% of women of reproductive age. The aetiology and pathogenesis of this disease remain unclear, and therefore, effective treatments are limited. Mouse models of endometriosis, while useful, do not model the complexity of the disease fully (Greaves *et al.* 2017). Gland organoids established from endometriotic lesions have been shown to differ from organoids derived in parallel from patient-matched eutopic endometrium, maintaining a heterogeneous disease profile, including aberrant signalling in integrin, PI3K-AKT, and WNT pathways, and thus identifying potential drug targets (Boretto *et al.* 2019). Furthermore, differences in glycodelin secretion mirror those in patients and controls (Luddi *et al.* 2020). However, this model is limited to only glandular epithelial cells. Sampson’s theory of retrograde menstruation proposes that endometriotic lesions derive from endometrial cells seeding the peritoneum at menstruation (Filby *et al.* 2020). Incorporating decidualised stromal cells from endometriotic lesions to generate endometriosis assembloids could provide a more encompassing model for lesion establishment and behaviour, while crossover experiments with healthy and pathological cell subpopulations could also aid in pinpointing the exact mechanisms for endometrial diseases.

Endometrial cancer is another potential candidate for organoid modelling since the pathology of endometrial cancer is largely unknown, and treatment is limited, mainly due to a lack of reliable preclinical models. Tumour-derived cell lines do not recapitulate clinical cancer heterogeneity, and genetic mouse models show aberrations inconsistent with clinical endometrial cancer (Boretto *et al.* 2019). Tumour-derived organoids from endometrial cancers have been shown to maintain clinical heterogeneity under long-term expansion but required medium optimisation to recapitulate the tumour niche, owing to non-cancerous organoids outcompeting cancerous organoids in the standard protocol (Boretto *et al.* 2019). This key observation highlights the necessity to consider the nuances of the local environment when developing future endometrial organoid-based disease models. Pre-cancerous organoid models have also been developed, such as organoids derived from hyperplastic endometrium, which faithfully reproduced the disease genotype. These organoids may provide a model that can be used to identify patient-specific biomarkers for early detection and intervention (Boretto *et al.* 2019).

The development of pathological endometrial organoid and assembloid models, in concert with healthy controls, provides promising tools to interrogate endometrial biology and pathology. In models that are physiologically or pathologically relevant, single-cell omics and functional assays may provide initial targets for gene editing. Owing to their clonal capacity, organoids provide an excellent candidate model for gene editing through CRISPR/Cas9 (Jinek *et al.* 2012, Cong *et al.* 2013, Mali *et al.* 2013) and this has been achieved in numerous organoid systems already, including the brain (Wang *et al.* 2015, Ogawa *et al.* 2018), liver (Artegiani *et al.* 2019), kidney (Freedman *et al.* 2015), and intestine (Matano *et al.* 2015, Roper *et al.* 2018). To our knowledge, only one study has demonstrated CRISPR/Cas9 in endometrial organoids (Chen *et al.* 2021), derived from mouse tissue, and therefore, efforts to advance the manipulation of human endometrial organoids will be welcomed. Genetic and gene regulatory manipulations will be essential for underpinning the key mechanisms and aberrations that lead to disease states, and as well as to open new avenues for biomarker investigation and drug discovery.

### Endometrial precision medicine, clinical trials, and therapeutic potential

The clearest benefit of patient-derived organoids and assembloids is to provide a novel opportunity to discover personalised treatments. Unlike previous models, patient-derived organoids have been demonstrated to maintain the heterogeneity and complexity of their clinical derivatives, allowing for the study of patient-specific phenotypes. Therefore, the next step in the development of pathological endometrial organoid models is to utilise them in preclinical drug screening tools. Organoids from other systems have already been used for drug screening. For example, cystic fibrosis (CF) is a genetic disorder caused by mutations in the cystic fibrosis transmembrane conductance regulator (CFTR) gene and causes particularly severe damage to the pulmonary and digestive systems (Dekkers *et al.* 2013). Clinical trials with CFTR-targeting drugs have shown variable efficacy among individuals. Organoids can be harnessed to target different mutations in the CFTR protein with the intention to discover personalised and effective treatments for CF (Dekkers *et al.* 2013, 2016, Berkers *et al.* 2019, Ramalho *et al.* 2021).

Endometrial organoids have already been used in pre-clinical drug screening studies for endometrial cancer (Chen *et al.* 2021). Unbiased drug screening of tumour organoids from mouse endometrial cancer identified MI-136 as a potential inhibitor of endometrial cancer through regulation of the HIF pathway, a novel mechanism distinct from those in acute myeloid leukaemia and prostate cancer (Chen *et al.* 2021). Additionally, the study reported that MI-136 also inhibited growth significantly in primary cancer organoids derived from patients (Chen *et al.* 2021). This provides evidence that endometrial organoids could be used as in pre-clinical drug screening studies.

To maximise the effective use of therapeutics, stratification of subjects in clinical trials is essential, especially in the case of a heterogeneous disease. Stratification and personalised medicine could be extrapolated to endometrial organoids and assembloids and the treatment of infertility. Our recent research has demonstrated that excessive decidual cellular senescence is associated with recurrent pregnancy loss (Lucas *et al.* 2020). Biomarkers for excessive decidual senescence could be utilised for screening for organoid- and assembloid-based pre-clinical drug interventions to reduce senescence and possibly reduce the burden of miscarriage. Only patients experiencing cycles with excessive senescence would likely see a benefit in reducing senescence, and therefore, it is essential to target this subset of patients. Primary endometrial stromal cells *in vitro* retain phenotypic characteristics of the donating patient. For example, endometrial stromal cells from women with recurrent pregnancy loss exhibit an imbalance of mature decidual and senescent decidual cells and an inability to recognise poor-quality embryos (Weimar *et al.* 2012, Lucas *et al.* 2020). Therefore, endometrial assembloid models should be able to mimic the behaviour of the tissue *in vivo* and provide a first step approach to patient-specific testing and stratification for treatment. Indeed, since organoid cultures can be bio-banked for future drug testing (Boretto *et al.* 2019), reducing the requirement for repeated biopsies, directed testing should be possible on specified cohorts of bio-banked samples.

Overall, organoids appear to be a promising model for drug discovery, pre-clinical evaluation, and stratification of subject groups for clinical trials and our challenge lie in driving the models from the fundamental study into the translational application to realise their full potential (Fig. 5).

## Conclusions

Organoid and assembloid models of the endometrium offer the opportunity to study embryo-endometrium interactions at the earliest timepoints in implantation, with increased physiological relevance, and thus present a promising tool for future research. Advancing our knowledge of the mechanisms and requirements for successful implantation also offers the prospect of identifying the routes through which the process fails, thus indicating possible therapeutic avenues. Through the ongoing development and characterisation of patient-specific organoid and assembloid models, targeted pre-conception therapies can be developed and tested extensively in pre-clinical studies. These exciting possibilities mean much work ahead: the full potential of organoid and assembloid models has not yet been realised, and the challenge now is to ensure that we drive the models forward to translational outcomes.

## Declaration of interest

E S L is an ordinary member of SRF council. The authors have no financial interests to declare.

## Funding

T M R is supported by a fellowship from the Warwick-Wellcome Trust Translational Partnership initiative. K M is funded by a Warwick Medical School scholarship. M T is funded by a EUTOPIA Co-tutelle PhD Scholarship between the University of Warwick and Vrije Universiteit Brussel (VUB). E S L is funded through a Wellcome Trust Investigator Award to Professor Jan Brosens (212233/Z/18/Z).

## Author contribution statement

T M R and E S L conceptualised the article. T M R, K M, M T, and E S L drafted the article. K M, M T, T M R, and E S L prepared the figures. E S L edited the article. All authors approved the final version.
